# Income-based inequalities in risk factors of NCDs and inequities of preventive care services amongst 202,682 adults: a cross-sectional study of South Asia Biobank

**DOI:** 10.1186/s12916-025-04308-3

**Published:** 2025-08-29

**Authors:** Bernardo Andretti, Petya Atanasova, Zoey Verdun, Nalinda Tharanga Wellappuli, Rajendra Pradeepa, Sudha Vasudevan, Akansha Tyagi, Ali Ahsan, Md. Mokbul Hossain, Abu Ahmed Shamim, Fahmida Akter, Sara Mahmood, Lathika Athauda, Manoja Gamage, Manuja Kaluarachchi, Thomas Burgoine, Soren Brage, Nita G. Forouhi, Ian Goon, Marie Loh, Prasad Katulanda, Anuradhani Kasturiratne, Khadija Irfan Khawaja, Sajjad Ahmad, Malay K. Mridha, Vinitaa Jha, Ranjit Mohan Anjana, John C. Chambers, Gary Frost, Franco Sassi, Marisa Miraldo

**Affiliations:** 1 Centre for Health Economics & Policy Innovation, Imperial Business School, South Kensington Campus, Exhibition Rd, London, SW7 2AZ UK; 2 Department of Economics and Public Policy, Imperial Business School, South Kensington Campus, Exhibition Rd, London, SW7 2AZ UK; 3 Department of Metabolism, Digestion and Reproduction, Faculty of Medicine, Faculty Building South Kensington Campus, Imperial College London, London, SW7 2AZ UK; 4 Madras Diabetes Research Foundation, Chennai, India; 5 Devki Devi Foundation, New Delhi, India; 6 Centre for Non-Communicable Diseases and Nutrition, BRAC James P Grant School of Public Health, BRAC University, Dhaka, Bangladesh; 7 Institute of Endocrinology and Metabolic Diseases, Services Institute of Medical Sciences, Lahore, Pakistan; 8 Faculty of Medicine, University of Kelaniya, Ragama, Sri Lanka; 9 Faculty of Medicine, University of Colombo, Colombo, Sri Lanka; 10 School of Public Health, Imperial College London, London, UK; 11 MRC Epidemiology Unit, Institute of Metabolic Science, School of Clinical Medicine, University of Cambridge, Cambridge, UK; 12 Tyree Foundation Institute of Health Engineering, UNSW, Sydney, NSW Australia; 13 Lee Kong Chian School of Medicine, Nanyang Technological University, Singapore, 308232 Singapore; 14 Department of Cardiology, Punjab Institute of Cardiology, Lahore, Pakistan

**Keywords:** Preventive health, Inequity, Inequality, Risk factors, Non-communicable diseases, South Asia

## Abstract

**Background:**

There is scant research examining income-based inequalities in risk factors of non-communicable diseases (NCDs) and inequities of preventive care services across the South Asian population.

**Methods:**

We conducted a cross-sectional study of 202,682 adults aged 18 or above in four South Asian countries: Bangladesh, India, Pakistan, and Sri Lanka. We combined South Asia Biobank (SAB) surveillance data with environmental mapping exposure and 24-h dietary recall to estimate income-based inequalities using concentration curves and concentration indices (CI) that measure the magnitude and directional inequality effects. We also computed the horizontal inequity index (HII) for need-standardised healthcare utilisation and advice by measuring the extent to which the distribution of health promotion advice matches the distribution of diet-related risk factor variables across the income distribution. We reported concentration index coefficients and standard errors.

**Results:**

Inequalities in exposure and diet-related risk factors of NCDs were observed. Underweight was concentrated amongst the poor (CI = − 0.16, SE = 0.005, *p* < 0.001), while overweight and obesity were concentrated amongst the rich (CI = 0.11, SE = 0.003, *p* < 0.001). Non-recommended intake of fats (CI = 0.04, SE = 0.003, *p* < 0.001) and carbohydrates were concentrated amongst the rich (CI = 0.05, SE = 0.003, *p* < 0.001), while non-recommended intake of free sugars (CI = − 0.05, SE = 0.004, *p* < 0.001) and fruits and vegetables amongst the poor (CI = − 0.07, SE = 0.005, *p* < 0.001). Exposure to unhealthy outlets was concentrated amongst the rich (CI = 0.02, SE = 0.002, *p* < 0.001). There were persistent and pro-rich inequities in healthcare utilisation (HII = 0.02, SE = 0.002, *p* < 0.001) and advice for salt reduction (HII = 0.02, SE = 0.004, *p* < 0.001), fat reduction (HII = 0.02, SE = 0.004, *p* < 0.001), healthy weight (HII = 0.03, SE = 0.006, *p* < 0.001), and fruits and vegetables consumption (HII = 0.04, SE = 0.004, *p* < 0.001).

**Conclusions:**

These findings indicate the need to address and mitigate income-based inequalities in diet-related risk factors of NCDs and underscore the need of policies directed at mitigating NCDs risk exposure and achieving improved and equitable access to healthcare.

**Supplementary Information:**

The online version contains supplementary material available at 10.1186/s12916-025-04308-3.

## Background

Diet-related risk factors contribute to the development of non-communicable diseases (NCDs). The exposure to the built food environment and its elements of *neighbourhood food environment*—i.e. the exposure to (un)healthy outlets around individuals—and *consumer food environment*—i.e. attributes experienced by consumers inside the food outlets such as availability, accessibility, price, and promotion [1–4]—have been associated with dietary outcomes [3] and are considered important “causes of the causes” of NCDs [1]. Modifiable risk factors associated with non-recommended diets, henceforth defined as diets above or below the recommended intake, such as excess levels of salt and insufficient levels of proteins, and metabolic risk factors, such as overweight and obesity, are important predictors of NCDs [5]. Crucially, risk factors can also vary across socioeconomic groups, being important contributors to inequalities in major NCDs [6].

To prevent the consequences of diet-related risk factors of NCDs, health promotion efforts should target its main causes and address the differential needs across population subgroups and their heterogeneity in environmental exposure [7]. Along these lines, improved healthcare could be promoted through preventive care services, a universal right by the World Health Organization (WHO) [8]. Preventive care is an important predictor of population health, mitigating individual’s risk of developing NCDs [9] and ultimately delaying the onset of illness and disease, thereby preventing deaths [10].

Despite the importance of preventive care for individuals’ health, and despite reducing health inequalities being listed by the WHO as a 2030 goal for countries, there is mounting evidence that its access is unequally distributed across the socioeconomic spectrum [11–13]. Previous studies have documented socioeconomic inequalities in healthcare utilisation across countries. However, most studies are focused on high-income countries and, critically, often focus on absolute inequalities in provision, without accounting for unequal population needs and therefore do not assess inequity. In South Asia, few studies explore inequality in access to healthcare. A recent study in India suggests that healthcare utilisation by individuals with cardiovascular disease is inequitably concentrated amongst individuals with higher income [13]. In Bangladesh, utilisation of facility-based delivery was concentrated amongst richer reproductive aged women [14]. In Pakistan, there was a pro-rich inequality of health human resources in doctors’ and nurses’ availability [15]. Notwithstanding, very few papers explore inequity [12] (i.e. unfair inequality), and even fewer assess the South Asian context [13]. The region faces key challenges in diet-related risk factors, being one of the world regions most affected by the dual burden of malnutrition [16] with high prevalence of both underweight and obesity [6]. South Asia also faces a fast-changing and evolving obesogenic food environment [17], thereby facing significant dietary intake challenges [3].

In this research, we characterised both income-based inequities in healthcare utilisation and advice across a sample of 202,682 individuals from Bangladesh, India, Pakistan, and Sri Lanka. To do so, we measured how different NCDs risk factors of (i) exposure to unhealthy/obesogenic food environments, (ii) non-recommended food intake, and (iii) weight-based needs are (un)equally distributed across the income distribution. We then used these metrics to create need-standardised healthcare utilisation and advice inequity indices [11, 12].

Our results can inform policymakers and practitioners. We underscore the importance of high-quality, universal coverage of primary care for the detection of NCDs [18] and the alleviation of the double burden of malnutrition [6, 16]. By providing universal and equitable access to preventive care, countries can mitigate income-based inequalities in health and achieve a healthier and fairer society [19]. Further, by identifying sources of inequalities in risk factors of NCDs, we highlight the importance of addressing their root causes by enacting effective food environment policies such as healthy food subsidies and restrictions on unhealthy food promotion [20].

## Methods

### Study design

In this cross-sectional study, we retrieved health surveillance data from November 2018 to December 2024 from the South Asia Biobank (SAB) [21]. This dataset provides individual level data including socio-demographic and health-related information based on health and lifestyle questionnaires, anthropometric measures, and individual geolocations of residence. We merged this dataset at the individual level with a 24-h recall of dietary intake using Intake24 [22] and assessed individuals’ diet-related needs. Finally, we merged these data with individual exposure to healthy and unhealthy food environments, using data from a ground truth survey mapping the type of food outlets in the neighbourhood of each participant residence [17].

### Participants

Our sample includes 202,682 individuals from four South Asian countries: Bangladesh, India, Pakistan, and Sri Lanka. Participants were recruited based on a cross-sectional, population-based adult sample from each country, extensively discussed elsewhere [17, 21]. Surveillance sites were centred on local primary community health care units. Governmental census data and household listings were used to map eligible participants. We recruited men and women of self-reported South Asian ethnicity, aged 18 years and above. We excluded women who were currently pregnant, non-permanent residents (for a year or more), people with serious illness expected to reduce life expectancy to less than a year, people who planned to leave the surveillance site within the next year, and people unable or unwilling to give informed consent.

### Study variables

#### Outcomes

Our primary outcome is a self-reported measure of healthcare utilisation. Using the SAB health and lifestyle questionnaire, participants were asked whether they had, during the past 3 years, visited a doctor or another healthcare worker (0 = no, 1 = yes). If participants responded yes, they were then asked if they had received healthcare advice on the following topics: (i) salt reduction, (ii) five servings of fruit and vegetable daily, (iii) fat reduction, (iv) sugar reduction, and (v) healthy and balanced weight maintenance (0 = no, 1 = yes).

#### Weight-based needs

To assess weight-based needs, we computed whether individuals were living with underweight (body mass index [BMI] < 18.5 kg/m^2^, 0 = no, 1 = yes), overweight (23 kg/m^2^ > BMI < 27.5 kg/m^2^, 0 = no, 1 = yes), or obesity (BMI > 27.5 kg/m^2^, 0 = no, 1 = yes), following the Asian BMI classification [23]. Following previous research [24], we also computed absolute distances to the “ideal” weight that avoid very high BMIs into a narrow *z* score range, and allow for more precise assessment (besides only using dichotomous variables) of weight-based needs. Amongst the underweight, we assessed the absolute linear differences between an individual’s BMI and the lower-bound for normal weight (18.5-BMI_i_ if underweight, 0 if normal weight), whereas amongst the overweight we computed a linear difference between individual BMI and the upper-bound for normal weight (23-BMI_i_ if overweight or obese, 0 if normal weight).

#### Environment risk factors

To assess the exposure to environmental risk factors, we constructed 500-m buffers around individuals’ residences. To measure exposure to obesogenic food environments, for each defined buffer, we calculated the density of healthy and unhealthy food outlets based on products sold within each food retailer, following international guidelines of Retail Food Environment Index (RFEI) [25] and the North American Industry Classification System (NAICS) [26]. Data collectors were instructed to code food outlets by type (e.g. supermarkets) and by six core food categories sold (e.g. sugar-sweetened beverages, fruits and vegetables). Healthy outlets were defined as (i) those selling *only* fruits and vegetables and (ii) supermarkets (except those which sold only confectionary, sugar-sweetened beverages, and sweet biscuits, which accounted for 49% of the supermarkets in our sample). All fast-food restaurants were defined as unhealthy. The outlets that did not sell fruits and vegetables, corner stores (except 7% that sold only fruits and vegetables), and all other outlet categories selling only confectionary, sugar-sweetened beverages, and sweet biscuits were also defined as unhealthy. Due to data collection challenges, not all geolocations were recovered, and 26.1% (*N* = 53,027) individuals had missing values on this variable (for details on missing geolocations, see Additional file 1: Sect. 1). See Additional file 1: Sect. 2 for detailed information on data collection procedure and outlet classification.

#### Diet-related needs

To assess diet-related needs, we have used Intake24 [22], a digital 24-h recall tool adapted for South Asia, to compute a series of variables that indicate non-recommended food intake, reflecting above and below levels of recommended intake (1 = below or above the recommended intake, 0 = recommended intake). We used the WHO Population Nutrient Intake Goals [27], recommended by the WHO Nutrient Profile Model for South-East Asia Region [28], as a reference to build these indicators. We calculated daily intake as a percentage of total daily energy intake. We computed non-recommended values for fats (below 15% or above 30% of total daily energy intake), saturated fats (above 10%), proteins (below 10% or above 15%), free sugars (above 10%), and carbohydrates (below 55% or above 75%). We also measured the total amount (in grams) of fruit and vegetable intake and used the threshold of 400 g for minimum adequate amount. Due to time taken for adaptation of the digital Intake24 tool for the South Asian context, data collection of dietary assessment started later (in 2020) than the overall study recruitment (in 2018). Some data collection also got disrupted during the COVID-19 pandemic. As such, a total of 116,431 participants filled in the Intake24 questionnaire (Additional file 1: Sect. 1).

#### Wealth

To compute inequalities and inequities, we ranked individuals based on their self-reported average per capita household monthly income in local currency units, adjusted by the purchasing parity power (PPP) across the four South Asian countries.

### Statistical analysis

We computed concentration curves, concentration indices, and horizontal inequity indices to assess income-based inequalities in risk factors of NCDs and inequities of utilisation of preventive care services in our sample. We considered as a cut-off of the *p* values < 0.05 and the absolute values of *t*-ratios > 1.96 for interpreting the significance of our findings.

#### Concentration curves

We used concentration curves to characterise socioeconomic inequalities in healthcare utilisation and advice and diet-related risk factors [29]. Concentration curves plot the cumulative proportion of the population ranked by the income variable against the cumulative proportion of healthcare utilisation and advice, weight-based needs, environmental risk factors, and diet-related needs. Concentration curves are compared to a 45-degree line (i.e. the equality line), which indicates perfect equality in the distribution of the variable of interest across the population. Whenever a concentration curve lies above (or below) the equality line, it represents that this variable is disproportionately concentrated amongst individuals of lower (higher) income [11, 29].

#### Concentration index (CI)

We further computed the degree (or intensity) and statistical significance of inequality by calculating, for each variable of interest, a concentration index (CI), which ranges between − 1 and 1 and is calculated as twice the area between the concentration curve and the equality line [11, 29, 30]. Negative (positive) and significant CI values suggest that the variable of interest is concentrated at the lower-(higher-)income individuals. CIs are used as our measure of *inequality*, i.e. absolute or raw differences between individuals across the income scale without adjustments for their needs. CIs across sex and countries are presented in Additional file 1: Sects. 3 and 4.

#### Horizontal inequity index

Following previous research [11, 12, 29, 30], we computed the horizontal inequity index (HII), which measures the degree of horizontal inequity in the distribution of healthcare utilisation across the income distribution by comparing the observed distribution of healthcare utilisation or advice by income with the distribution of the health-related need variables. While inequalities refer to differences in healthcare utilisation and advice or any “need” variables between individuals of different incomes, inequity refers to whether those differences decrease, remain, or increase after adjustments for individuals’ health-related needs [11, 12]. We utilise an indirect standardisation method to adjust for the need variables, and estimate a health regression as follows, using probit given the binary nature of our dependent variables [11, 12]:

$${y}_{i}=\alpha +{\sum }_{j}{\beta }_{j}{x}_{ji}+{\sum }_{k}{\gamma }_{k} {z}_{ki}+{\varepsilon }_{i}$$

in which ${y}_{i}$ is healthcare utilisation or advice (on each of the topics) received by each individual, ${x}_{j}$ are the need variables of risk factors (i.e. weight-based, environmental, and diet-related needs) and age and sex dummies, and ${z}_{k}$ are the non-need variables of country fixed-effects and month-year fixed-effects. We then obtain parameter estimates, $\widehat{\alpha }, {\widehat{\beta }}_{j}, {\widehat{\gamma }}_{k}$, individual level need variables (${x}_{ji}$), and sample means of non-need variables (${\overline z}_k$). Therefore, we can obtain the predicted values for healthcare utilisation and advice:

$$\widehat y_i^X=\widehat\alpha+\sum\nolimits_j{\widehat\beta}_jx_{ji}+\sum\nolimits_k{\widehat\gamma}_k{\overline z}_k\\$$

We then obtain estimates of the indirectly standardised healthcare utilisation (or advice) by computing the difference between actual and expected healthcare utilisation and advice (*x*), plus the overall sample mean:

$${\widehat y}_i^{IS}=y_i-{\widehat y}_i^X+\overline y$$

By equalising needs for individuals based on the average relationship between need and treatment for the population as a whole (which serves as the vertical equity “norm”), we can assess deviations from this norm across income [12]. In other words, the HII obtained is the concentration index of the need-standardised healthcare utilisation and advice. Significant and positive HII results represent pro-rich inequity, significant and negative results pro-poor inequity, and null results equity. HIIs across sex and countries are presented in Additional file 1: sections 5, 6, and 7.

## Results

### Descriptive statistics

Table 1 displays descriptive statistics of our sample of 202,682 individuals distributed across four South Asian countries: Bangladesh (*N* = 70,556), India (*N* = 31,644), Pakistan (*N* = 49,059), and Sri Lanka (*N* = 51,423). While 47.1% of the lowest-income individuals visited healthcare workers in the previous 3 years, 55.4% of the highest-income ones did so (*p* < 0.001). Females visited healthcare workers to a greater extent, compared to males (51.9% vs. 45.6%, *p* < 0.001). Underweight was more prevalent amongst the lowest-income individuals (12.1%) while obesity was more prevalent amongst the third-income quartile (31.6%). Non-recommended intake of different foods was prevalent across sex and countries. Exposure to unhealthy food outlets was also much more prevalent than to healthy food outlets across all country and sex subgroups.

**Table 1:** Descriptive statistics of participants in South Asia Biobank (SAB) on healthcare utilisation and advice and risk factors of NCDs

	**Total**	**Country**				**Income quartiles**				**Sex**	
		**Bangladesh**	**India**	**Pakistan**	**Sri Lanka**	**1st quartile**	**2nd quartile**	**3rd quartile**	**4th quartile**	**Male**	**Female**
**Healthcare utilisation and advice**											
**Visited healthcare worker (*N*, %)**	100,297 (49.5%)	36,034 (51.1%)	17,165 (54.2%)	14,048 (28.6%)	33,050 (64.3%)	20,794 (47.1%)	23,176 (51.4%)	24,113 (43.6%)	32,214 (55.4%)	35,809 (45.6%)	64,496 (51.9%)
Advice for salt reduction (*N*, %)	37,309 (18.4%)	13,913 (19.7%)	4906 (15.5%)	8217 (16.7%)	10,273 (20.0%)	8778 (19.9%)	8131 (18.0%)	8490 (15.4%)	11,910 (20.5%)	13,059 (16.6%)	24,250 (19.5%)
Advice for fruits and vegetables intake (*N*, %)	48,413 (23.9%)	21,548 (30.5%)	7652 (24.2%)	8484 (17.3%)	10,729 (20.9%)	10,994 (24.9%)	10,884 (24.1%)	11,001 (19.9%)	15,534 (26.7%)	16,978 (21.6%)	31,439 (25.3%)
Advice for fat reduction (*N*, %)	41,684 (20.6%)	14,665 (20.8%)	6433 (20.3%)	8048 (16.4%)	12,538 (24.4%)	9268 (21.0%)	9369 (20.8%)	9558 (17.3%)	13,489 (23.2%)	14,712 (18.7%)	26,973 (21.7%)
Advice for healthy weight (*N*, %)	28,559 (14.1%)	9156 (13.0%)	4347 (13.7%)	6726 (13.7%)	8330 (16.2%)	6013 (13.6%)	6160 (13.7%)	6558 (11.9%)	9828 (16.9%)	9432 (12.0%)	19,129 (15.4%)
Advice for sugar reduction (*N*, %)	28,069 (13.8%)	7360 (10.4%)	4122 (13.0%)	6537 (13.3%)	10,050 (19.5%)	6293 (14.3%)	6207 (13.8%)	6347 (11.5%)	9222 (15.9%)	10,026 (12.8%)	18,044 (14.5%)
**Weight-based needs**											
Underweight (*N*, %)	17,148 (8.5%)	8951 (12.7%)	1697 (5.4%)	2554 (5.2%)	3946 (7.7%)	5331 (12.1%)	4209 (9.3%)	4004 (7.2%)	3604 (6.2%)	7907 (10.1%)	9243 (7.4%)
Distance to underweight^a^ (mean, SD)	0.309 (0.800)	0.300 (0.742)	0.298 (0.824)	0.324 (0.835)	0.322 (0.877)	0.385 (0.884)	0.308 (0.803)	0.290 (0.791)	0.248 (0.700)	0.276 (0.731)	0.339 (0.856)
Overweight and obesity (*N*, %)	125,435 (61.9%)	32,184 (45.6%)	23,174 (73.2%)	37,619 (76.7%)	32,458 (63.1%)	23,868 (54.1%)	26,094 (57.9%)	36,103 (65.3%)	39,370 (67.7%)	42,130 (53.7%)	83,318 (67.1%)
Obesity (*N*, %)	57,262 (28.3%)	9213 (13.1%)	11,684 (36.9%)	23,414 (47.7%)	12,951 (25.2%)	10,682 (24.2%)	11,070 (24.5%)	17,452 (31.6%)	18,058 (31.0%)	15,043 (19.2%)	42,226 (34.0%)
Distance to overweight^b^ (mean, SD)	3.394 (4.175)	1.780 (2.672)	4.141 (4.242)	5.487 (5.214)	2.976 (3.567)	3.056 (4.176)	2.995 (3.908)	3.743 (4.388)	3.605 (4.118)	2.414 (3.360)	3.995 (4.499)
**Diet-related needs**											
Non-recommended fat (*N*, %)	60,343 (51.8%)	19,065 (52.6%)	6698 (57.4%)	14,274 (46.9%)	20,306 (53.3%)	12,202 (50.4%)	13,219 (51.4%)	16,516 (50.9%)	18,406 (54.0%)	22,696 (50.8%)	37,655 (52.5%)
Non-recommended saturated fat (*N*, %)	44,844 (38.5%)	9350 (25.8%)	4678 (40.1%)	13,103 (43.1%)	17,713 (46.5%)	8792 (36.3%)	9587 (37.3%)	12,342 (38.0%)	14,123 (41.4%)	16,347 (36.6%)	28,505 (39.7%)
Non-recommended free sugars (*N*, %)	24,799 (21.3%)	2011 (5.6%)	4469 (38.3%)	8379 (27.6%)	9940 (26.1%)	5221 (21.6%)	5651 (22.0%)	7348 (22.6%)	6579 (19.3%)	9196 (20.6%)	15,606 (21.7%)
Non-recommended proteins (*N*, %)	57,573 (49.4%)	21,078 (58.2%)	5850 (50.1%)	13,610 (44.8%)	17,035 (44.7%)	12,484 (51.6%)	12,717 (49.5%)	15,805 (48.7%)	16,567 (48.6%)	22,153 (49.6%)	35,423 (49.4%)
Non-recommended fruits and vegetables (*N*, %)	99,412 (85.4%)	28,814 (79.5%)	10,441 (89.4%)	26,754 (88.0%)	33,403 (87.6%)	20,979 (86.6%)	22,095 (86.0%)	28,075 (86.5%)	28,263 (82.9%)	36,891 (82.5%)	62,535 (87.2%)
Non-recommended carbohydrates (*N*, %)	49,966 (42.9%)	16,017 (44.2%)	5700 (48.8%)	11,785 (38.8%)	16,464 (43.2%)	10,014 (41.4%)	10,764 (41.9%)	13,614 (42.0%)	15,574 (45.7%)	18,909 (42.3%)	31,064 (43.3%)
**Environment exposure needs**^**c**^											
Healthy food outlets density (mean, SD)	0.117 (0.132)	0.052 (0.084)	0.201 (0.079)	0.195 (0.145)	0.119 (0.147)	0.103 (0.124)	0.107 (0.128)	0.134 (0.134)	0.119 (0.136)	0.111 (0.131)	0.121 (0.132)
Unhealthy food outlets density (mean, SD)	0.617 (0.327)	0.798 (0.256)	0.632 (0.148)	0.258 (0.192)	0.605 (0.337)	0.652 (0.323)	0.662 (0.313)	0.563 (0.328)	0.609 (0.330)	0.635 (0.326)	0.606 (0.327)
*N*	202,682 (100%)	70,556 (35%)	31,644 (16%)	49,059 (24%)	51,423 (25%)	44,131 (22%)	45,101 (22%)	55,280 (27%)	58,170 (29%)	78,469 (39%)	124,230 (61%)

^a^Absolute linear differences between an individual’s BMI and the lower-bound for normal weight (18.5-BMI_i_ if underweight, 0 if normal weight)

^b^Absolute linear differences between individual BMI and the upper-bound for normal weight (23-BMI_i_ if overweight or obese, 0 if normal weight)

^c ^Outlets were originally categorised as healthy, unhealthy, and “other”. We use healthy and unhealthy only, as we cannot ascertain the impact of the “other” category as a health risk

### Assessing inequalities: concentration curves and concentration indices (CI) of healthcare utilisation, advice, and diet-related needs

**Fig. 1:**
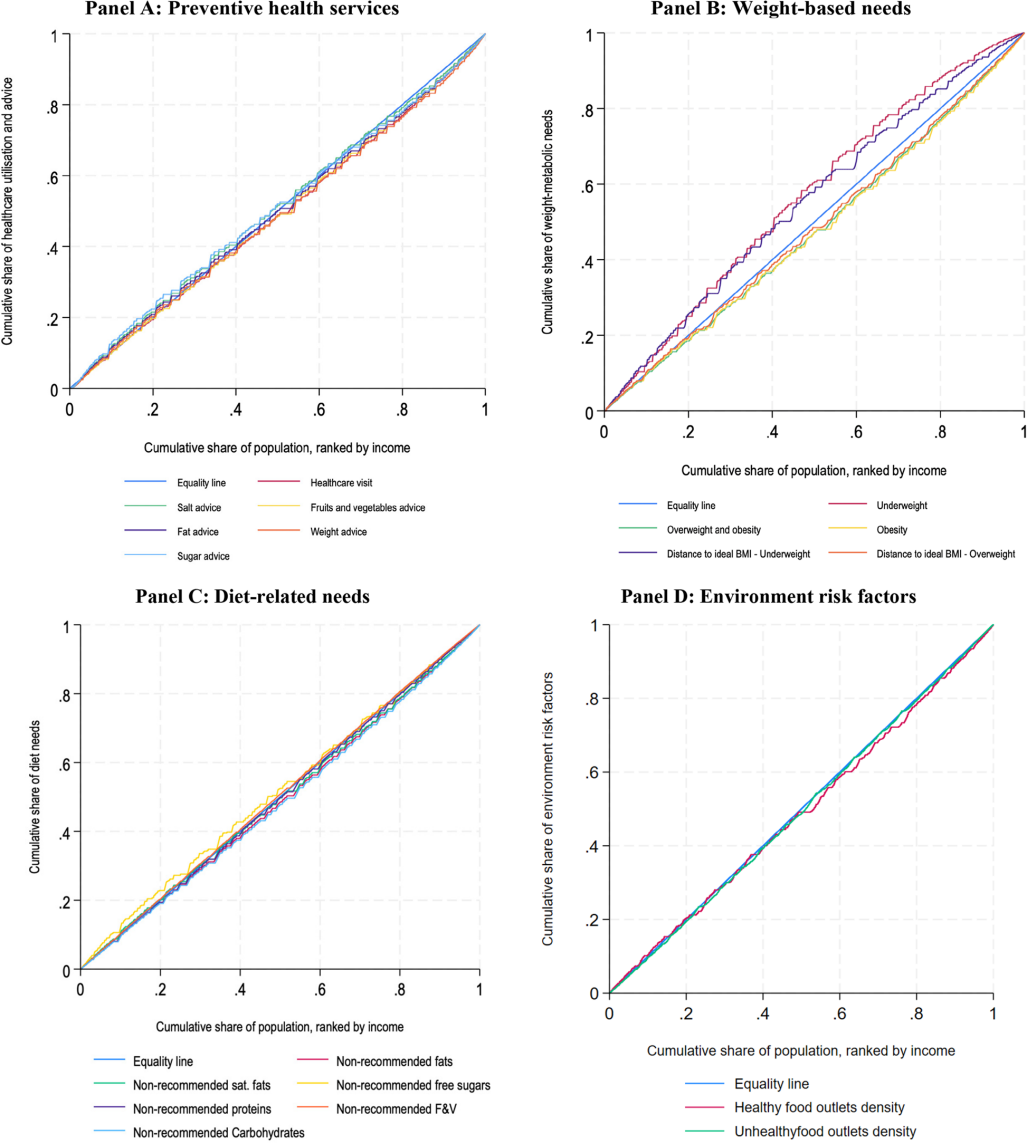
Concentration curves for healthcare utilisation, advice, and risk factors of non-communicable diseases. Note: concentration curves plot the cumulative proportion of the population ranked by the income variable against the cumulative proportion of each variable of interest. Every graph contains a 45-degree line (i.e. equality line, blue) that represents equal distribution across income. Whenever a concentration curve lies above (or below) the equality line, it represents that this variable is disproportionately concentrated amongst individuals of lower (higher) income. BMI refers to body mass index

Figure 1 plots the concentration curves for all the variables included in the analysis, and Table 2 shows the concentration indices for all relevant variables. Healthcare utilisation was concentrated amongst the rich (i.e. below the equality line, CI = 0.05, *p* < 0.001), as well as most healthcare advice except for salt and sugar, which was slightly concentrated amongst the poor (Fig. 1A). Figure 1B displays weight-based needs. Underweight was concentrated amongst the poor (CI = − 0.16, *p* < 0.001), while overweight and obesity (CI = 0.11, *p* < 0.001) and obesity (CI = 0.06, *p* < 0.001) were concentrated amongst the rich. In Fig. 1C, diet-related needs of fats (CI = 0.04, *p* < 0.001), saturated fats (CI = 0.01, *p* = 0.023), and carbohydrates (CI = 0.05, *p* < 0.001) were slightly concentrated amongst the rich, whereas non-recommended free sugars (CI = − 0.05, *p* < 0.001) and fruit and vegetable intake (CI = − 0.07, *p* < 0.001) were slightly concentrated amongst the poor. Finally, Fig. 1D shows that the share of both healthy (CI = 0.02, *p* < 0.001) and unhealthy outlets (CI = 0.02, *p* < 0.001) were slightly concentrated amongst the rich.

**Table 2:** Concentration indices of healthcare utilisation and advice and diet-related needs

	*N*	CI	SE	*p* value^b^
**Healthcare utilisation and advice**				
**Visited healthcare worker**	202,682	0.046	0.003	0.000
Advice for salt reduction	202,682	− 0.023	0.003	0.000
Advice for fruits and vegetables intake	202,682	0.023	0.003	0.000
Advice for fat reduction	202,682	0.002	0.003	0.460
Advice for healthy weight	202,682	0.025	0.004	0.000
Advice for sugar reduction	78,467	0.037	0.006	0.000
**Weight-based needs**				
Underweight	202,682	− 0.158	0.005	0.000
Distance to underweight^a^	77,247	− 0.119	0.005	0.000
Overweight and obesity	202,682	0.107	0.003	0.000
Obesity	202,682	0.060	0.003	0.000
Distance to overweight^a^	184,725	0.024	0.002	0.000
**Diet-related needs**				
Non-recommended fat	116,431	0.041	0.003	0.000
Non-recommended saturated fat	116,431	0.008	0.003	0.023
Non-recommended free sugars	116,431	− 0.046	0.004	0.000
Non-recommended proteins	116,431	0.006	0.003	0.060
Non-recommended fruits and vegetables	116,431	− 0.074	0.005	0.000
Non-recommended carbohydrates	116,431	0.051	0.003	0.000
**Environment exposure needs (shares of 500 m)**				
Healthy food outlets^a^	149,698	0.016	0.002	0.000
Unhealthy food outlets^a^	149,698	0.022	0.002	0.000

Note: we used Wagstaff concentration indices for bounded variables, with fixed scales, and limits from 0 to 1, in which zero corresponds to a situation of complete absence (e.g. no healthcare visits)

^a^We computed the standard concentration index with a zero-fixed lower limit

^b^We used the cut-off of *p* < 0.05 to interpret the significance of our findings

We also observed important differences across sex (Additional file 1: Table S1). Healthcare utilisation was concentrated amongst the rich for both male and female participants. However, while richer males had disproportionate access to healthcare advice across all indicators, advice for salt, fat reduction, and sugar were concentrated amongst the poor for females. Despite having the same direction, coefficients for underweight, overweight, and obesity were higher in magnitude for males compared to females. No substantial differences were observed in environmental and diet-related needs.

Finally, we observed differences across countries (Additional file 1: Table S2). Healthcare utilisation and advice were always concentrated amongst the rich in Bangladesh and India but concentrated amongst the poor in Pakistan and Sri Lanka (except for advice for healthy weight in Sri Lanka). Prevalence of underweight (overweight and obesity) was concentrated amongst the poor (the rich) across all countries. Dietary inadequacies showed overall a similar trend across countries. Finally, the density of unhealthy food outlets was concentrated amongst the poor in Bangladesh and Pakistan only.

### Assessing inequities: horizontal inequity index (HII) of healthcare utilisation and advice

Table 3 shows the predicted probability and actual usage of preventive care services. The actual distribution observed was pro-rich (model 1), and the need-expected distribution was pro-poor (model 2). This need-expected distribution is a result of the fact that “need”, as proxied by demographic, diet-related, environmental, and morbidity characteristics, was concentrated amongst lower-income groups. As a result, for the second lower-income quintile of individuals, the probability of reporting a preventive care contact was 3.4% lower than would be expected on average given their need, whereas the highest-income 20% of individuals reported a probability of such a contact that was 6.6% higher than expected. It is therefore no surprise that the need-standardised distribution showed an even more pro-rich distribution than the actual distribution (model 3). Table 1 also depicts the horizontal inequity index of healthcare utilisation, adjusted for risk factors of NCDs. It shows that healthcare utilisation was inequitably concentrated amongst higher-income individuals, even more so when adjusting for the core need variables (HII = 0.02, SE = 0.002, *p* < 0.001). Subgroup analyses show that after adjusting for risk factors of NCDs, the HII was concentrated amongst the higher-income individuals for both males and females (Additional file 1: Tables S3 and S4) and for Bangladesh (Additional file 1: Table S5) and Pakistan (Additional file 1: Table S7). HIIs were not significant for either India (Additional file 1: Table S6) or Sri Lanka (Additional file 1: Table S8), suggesting equitable distribution in those countries.

**Table 3:** Predicted probability and actual usage of preventive care in the past 3 years (*N* = 88,208)

Income quintile	Actual (Model 1)	Probit		Need-standardised
		Need-predicted (Model 2)	Difference = predicted − actual	Probit estimates (Model 3)
Poorest 20%	0.489	0.468	2.10%	0.477
2nd poorest 20%	0.422	0.456	− 3.39%	0.422
Middle	0.439	0.454	− 1.46%	0.441
2nd richest 20%	0.431	0.447	− 1.62%	0.439
Richest 20%	0.518	0.453	6.56%	0.521
Mean	0.459	0.455	0.40%	0.460
**Horizontal inequity index (HII)**	0.010	− 0.007		0.017
SE	0.002	0.0003		0.002
*t*-ratio^a^	4.118	− 23.557		7.237

Note: model 1 displays the actual usage of preventive care services; model 2 displays the need-predicted usage of healthcare services, using (i) weight-based needs, (ii) environment risk factors, (iii) dietary needs, and (iv) sex and age differences; model 3 displays the need-standardised probit estimates of predicted preventive care, adjusted by the need variables. All models show actual and predicted values across income quintiles and the mean values. The horizontal inequity index represents the adjusted concentration indices of each distribution

^a^We used the cut-off of *t* > 1.96 to interpret the significance of our findings

Table 4 depicts the HII of healthcare advice. It shows positive and significant HIIs for salt reduction advice (HII = 0.02, *p* < 0.001), fruit and vegetables consumption advice (HII = 0.04, *p* < 0.001), fat reduction advice (HII = 0.02, *p* < 0.001), and healthy weight maintenance advice (HII = 0.03, *p* < 0.001), which means that healthcare advice in those variables was inequitably (and unfairly) concentrated amongst higher-income individuals. Sugar reduction advice, on the other hand, was equitably distributed, i.e. access reflects need across the socioeconomic gradient. Results across sex and countries are presented in Additional file 1: Tables S9–S13.

**Table 4:** Horizontal inequity index (HII) for healthcare advice (*N* = 88,208)

Advice towards…	Horizontal inequity index (HII)	SE	*t*-ratio^a^
Salt reduction	0.016	0.004	3.565
Fruits and vegetables consumption	0.037	0.004	8.813
Fat reduction	0.022	0.004	5.014
Healthy weight	0.026	0.006	4.738
Sugar reduction	− 0.002	0.006	− 0.358

Note: the horizontal inequity index (HII) represents the concentration indices of each healthcare advice, need-standardised

^a^We used the cut-off of *t* > 1.96 to interpret the significance of our findings

## Discussion

We relied on a unique dataset across four South Asian countries (Bangladesh, India, Pakistan, and Sri Lanka) [21] to advance the current literature and offer an assessment of income-based inequities in preventive care services. We show that, after adjusting for key risk factors of NCDs, higher-income individuals have a disproportionate access to preventive care services, a result corroborated by previous research [13, 14]. We also show that, despite having a lower prevalence of disease [6], inequities are stronger for men (vs. women) and that inequities are present in Bangladesh and Pakistan, but not in India or Sri Lanka. Our findings also indicate that there are important income-based inequalities in such risk factors. Lower-income individuals have a much higher prevalence of underweight, but a lower level of overweight and obesity, suggesting that the double burden of malnutrition is not equally distributed across the socioeconomic gradient [17]. We also found a higher exposure to unhealthy food outlets amongst the wealthy and important differences in dietary intake across the income distribution.

Our work adds to a scant yet growing research on inequalities and inequities in healthcare utilisation in South Asia, a region facing significant challenges of rapid urbanisation and deep inequalities in primary healthcare [31, 32]. Our results are in line with previous research in the region that documents important income-related inequalities in the distribution of healthcare professionals across districts in Pakistan [15], inequalities in facility delivery utilisation amongst reproductive-age women in Bangladesh [14], and inequities in healthcare utilisation amongst individuals with cardiovascular disease in India [13]. Our study builds on that evidence by documenting income-based inequities in healthcare utilisation and income-based inequalities in health-related needs.

Our study provides important policy implications. By showing that higher-income households have disproportionated access to preventive care, our findings underscore the necessity of ensuring equitable access for all individuals, following recommendations from the WHO, the World Bank, and the Organisation for Economic Co-operation and Development (OECD) [19]. Broad approaches suggested by WHO are still relevant and promising in that respect: (i) target healthcare programmes for disadvantaged populations—focusing on health literacy, promotion, and access, (ii) close health gaps between worse-off and better-off groups, and (iii) address the social health gradient across the whole population [8]. By ensuring equitable healthcare utilisation and advice across the socioeconomic gradient, policymakers can help reduce inequities and prevent the development of NCDs.

Our findings also suggest that there are a series of actions targeted at decreasing diet-related risk factors that may be promising to tackle inequities. Policymakers play a pivotal role in developing strategies to encourage affordable and healthy diets, which can alleviate root causes of NCDs by mitigating environmental risk exposure [20]. A recent study documents that while Bangladesh, India, Pakistan, and Sri Lanka have multisectoral action plans to prevent and control NCDs and have set time bound targets on NCDs risk factors and management, there has been limited progress towards the prevention of unhealthy diets and unhealthy food environments [20]. Therefore, governments in this region should focus on ensuring that all individuals have equitable access and availability of healthy, affordable, and nutrient-dense foods [27]. To do so, effective actions may include targeting the food environment to reduce unhealthy food intake and promote healthy diets [33] and introducing food taxes (e.g. on products high in fat and sugar) and subsidies (e.g. on fruits and vegetables) to encourage healthier eating across the population, as well as restricting unhealthy food promotion and availability [20]. Furthermore, while the assessed countries have mostly focused on the prevention of undernutrition and food insecurity, our results highlight the need for more targeted interventions to reduce underweight prevalence amongst the disadvantaged and to reduce overweight and obesity amongst the wealthier. While some of the interventions (such as taxes and restrictions) could be more efficient in reducing unhealthy intake and alleviating obesity, others (such as subsidies) could make healthy eating more affordable and be more efficient in reducing underweight. Thus, our results suggest that one-size-fits-all interventions would not suffice to reduce ubiquitous health inequalities. Nevertheless, future studies should test the effectiveness and feasibility of different interventions to be implemented.

Our study has several limitations. We rely on a cross-sectional dataset, and we are thus limited to assessing correlations, without the ability to make any causal claims. Despite utilising a unique dataset, we rely on self-reported outcomes of healthcare utilisation and advice and cannot ascertain whether participants visited healthcare workers. Our findings cannot speak to the quality of healthcare services being provided, an important and often overlooked dimension of preventive care services [19]. Future research should address how effective preventive care provision and advice is across the South Asian countries. When measuring the food environment, we do not capture important exposures to their food environment (e.g. work and commute) and cannot speak to how influential the food environment around homes is to those households. When measuring diet, we used a validated tool (Intake24) that, like other dietary assessment methods, comes with the limitations of potential bias in which the true dietary intake is unknown [22]. However, we were careful to ensure that trained data collectors conducted the survey, and we conducted data quality and internal validation checks. Furthermore, our indicators of non-recommended intake were constructed based on general WHO recommendations [27]. Despite being recommended by the WHO for South Asian contexts [28], we acknowledge the limitation as a diversified and balanced healthy diet depends not only on context and culture, but also on individual needs. Although BMI is used in this study as an indicator of individuals’ health, we acknowledge that it is a limited indicator in South Asia, as previous evidence suggests that BMI might not predict cardiovascular diseases and mortality in the region for all groups of population we use in our study [34]. Finally, our cross-country results warrant caution, as they might reflect different sampling strategies, which can reflect, in practice, in not directly comparable samples (e.g. more vs. less urbanised sites). Our results across sex also warrant some caution, as we cannot capture differential exposures to environment between men and women (e.g. time staying at home). Future research can further advance the methodologies outlined here and link administrative healthcare data to individuals’ diet-related risk factor exposure on a longitudinal study.

## Conclusions

Despite these limitations, the current research provides novel evidence on important and persistent health inequalities and inequities in an understudied population by relying on a unique dataset of four South Asian countries. In the South Asian context of growing urbanisation, it is expected an increase in the exposure to unhealthy obesogenic environments and a growth of purchasing power, which can further exacerbate risk factors of NCDs. Therefore, our study provides evidence for policymakers to propose timely action in at least two fronts: ensuring equitable access to preventive care and placing efforts into ensuring a healthier food environment for all.

## Supplementary Information

Additional file 1. Income-based inequalities and inequities.

## Data Availability

Surveillance and Intake24 data are available to researchers upon request to the study Data Access Committee. For further information about the South Asia Biobank study please contact manuja.kaluarachchi@imperial.ac.uk. For the surveillance data, contact forms and emails are provided on the GHRU website (https://www.ghru-southasia.org). For the environmental data, requests should be made via email to health.economics@imperial.ac.uk.

## Abbreviations

BMI: Body mass Index
CI: Concentration Index
HII: Horizontal Inequity Index
Intake24: 24-H recall of dietary intake
NAICS: North American Industry Classification System
NCDs: Non-communicable Diseases
OECD: Organisation for Economic Co-operation and Development
PPP: Purchasing parity power
RFEI: Retail Food Environment Index
SAB: South Asia Biobank
WHO: World Health Organization
