# Supplementary material for: Income-based inequalities in risk factors of NCDs and inequities of preventive care services amongst 202,682 adults: a cross-sectional study of South Asia Biobank
Source: BMC Med. 2025 Aug 29;23:504. doi: 10.1186/s12916-025-04308-3 (PMC12395800; doi:10.1186/s12916-025-04308-3)
Supplement: Supplementary file 1 — Additional file 1. Income-based inequalities and inequities. [file 12916_2025_4308_MOESM1_ESM.docx]

Additional file for

**Income-based inequalities in risk factors of NCDs and inequities of preventive care services among 202,682 adults: a cross-sectional study of South Asia Biobank**

Table of contents

[**1.** **Missing data information** 2](#_Toc204702656)

[**2.** **Environmental Mapping information** 3](#_Toc204702657)

[**3.** **Concentration indices (CI) of healthcare utilisation and advice, and diet-related needs across sex** 5](#_Toc204702658)

[**4.** **Concentration indices (CI) of healthcare utilisation and advice, and diet-related needs across countries** 6](#_Toc204702659)

[**5.** **Horizontal inequity index (HII) of healthcare utilisation across sex, using Probit** 7](#_Toc204702660)

[**6.** **Horizontal inequity index (HII) of healthcare utilisation across countries, using Probit** 8](#_Toc204702661)

[**7.** **Horizontal inequity index (HII) of healthcare advice across sex and countries, using Probit** 10](#_Toc204702662)

# **Missing data information**

In this section, we detail the main data gaps of our dataset. All participants filled in the questionnaire with key sociodemographic information, as well as the health and lifestyle questionnaire. All participants also filled in all information about healthcare utilisation and advice and had their weight-based needs measured at data collection. However, due to issues in the data collection procedure, information on geolocations and dietary recall were not obtained for a subset of participants.

**Geolocations.** 26.15% of individuals in the sample have missing geolocations (N = 53,027). Across countries, missing information are distributed as follows: India has the highest percentage of missing geolocations (N = 12,713, 40.67%), followed by Pakistan (N = 17,126, 34.82%), Sri Lanka (N = 17,212, 33.42%), and Bangladesh (N = 5,976, 8.43%).

For most participants, geolocation was recorded at the time of data collection, where data collectors went to participants’ residents and recorded their precise location. Due to the onset of the COVID-19 pandemic, however, some participants were invited to local clinics, or approached via other methods, for data collection. This in turn resulted in a greater difficulty to precisely assess some individual’s geolocations.

**Dietary Recall (Intake24).** 42.55% of individuals in the sample have missing on Intake24 (N = 86,306). Across countries, missing information is distributed as follows: India has the highest percentage of missing Intake24 (N = 19,835, 63.45%), followed by Bangladesh (N = 34,478, 48.65%), Pakistan (N = 18,669, 37.96%), and Sri Lanka (N = 13,324, 25.87%).

To conduct dietary recall in our sample, a huge effort was undertaken to adapt the digital 24h recall tool. Therefore, while the main baseline data collection started in 2018, Intake24 data collection started only later, in 2020. SAB baseline data collection commenced in November 2018, initially without collection of dietary data as the choice of dietary assessment method had not been determined. Due to the scale of SAB data collection and for reasons of feasibility, time and cost, the aim was to identify a digital tool using a proven dietary assessment method that could enable efficient data collection with electronic data capture and auto-coding of nutrient intakes. No such tool was available in ready-state for use in the South Asia setting, however after an options appraisal, the 24-hour dietary recall tool, Intake24, a web-based dietary assessment instrument based on the multiple pass recall method, was identified as offering good potential for development. Prior to implementation, the tool required significant adaptation to compile and configure food databases suitable for the SAB setting, to enable participants to search for and identify the foods and drinks they had consumed. The Intake24 system had been used extensively for population health research in the UK, and at the time of consideration for SAB, it was being updated for implementation in the UK National Diet and Nutrition Survey. Intake24 was selected as the dietary method for SAB towards the end of 2018. System development and adaptation (including deployment of an ‘offline’ option for local data collection in areas of poor internet connectivity) was undertaken during 2019, led by Cambridge University. Data collection with Intake24 (Software Version 3: Intake24 [South Asia] Cambridge 2019) was first introduced in SAB in January 2020 for collection of one dietary recall per participant, generally obtained at the end of the clinic visit.

For SAB field data collection, Intake24 was deployed online (web-served from Cambridge, UK) and via locally-served instances of the system in areas of poor internet connectivity at SAB clinic sites. Data collection was administered by SAB interviewers (using tablets) for collection of a single individual-level 24-hour dietary recall from all participants who were willing to complete the Intake24 component. Once Intake24 was available and implemented at respective clinic sites, interviewers completed the recall generally at the end of the SAB clinic visit using the SAB data collection tablet. Participants were able to view the display screen, relevant particularly for collecting portion information as the tool uses a range of portion estimates including visual guide and as-served images. Interviewers verbally translated instructions, questions and food descriptions from English into regional languages to facilitate recall administration as required. In brief, completion of a dietary recall using Intake24 for SAB was as follows. The participant (via the interviewer) was taken through a series of prompts to report and quantify all food and drink consumed in the preceding 24-hours. They provided detail by answering a number of food related and general questions. Participants were first guided to enter everything they had to eat (as separate foods and drinks) at each eating occasion including all meals and snacks. Next, they were asked to match each food/drink they have entered to foods and drinks in the Intake24 food database. After they had matched the food, they were asked to describe how much they had consumed for each food listed using a range of portion estimation methods offered according to the specific food. Where interviewers were unable to identify or match a participant’s food to the embedded food list in Intake24, they were able to report missing foods via the tool. Missing foods were later manually coded for inclusion in the dietary intake data.

Whilst clinic data collection in SAB commenced at the end of 2018, dietary data collection was introduced from January 2020 first in Sri Lanka and subsequently in Bangladesh, India (September 2020) and Pakistan (October 2020). Due to the global COVID-19 pandemic, data collection in each study region was interrupted at different time points. For preparation of the dietary data ahead of release, all missing foods were manually coded, and a series of more comprehensive data quality checks were carried out on the dietary data.

**Combining Geolocations and Dietary Recall**. 56.48% of individuals in the sample have missing observations on either geolocation or dietary recall (N = 114,552). Across countries, missing information is distributed as follows: India has the highest percentage of missing information (N = 26,169, 83.72%), followed by Pakistan (N = 26,301, 53.48%), Bangladesh (N = 36,587, 51.63%), and Sri Lanka (N = 25,495, 49.50%).

Table S1 presents key characteristics of individuals with missing diet and geolocation and participants without any missing information.

# **Environmental Mapping information**

- 1. ***Data collection procedure***

Data was retrieved from a survey protocol in Kobo Toolbox application (www. kobotoolbox.org), used as the study instrument. Questions included geolocations (automatically retrieved), type of food outlets (e.g., FV, confectionary, and fast food). Questions and data collection procedure were adapted from the International Network for Food and Obesity/NCDs Research, Monitoring and Action Support (www.informas.org) and Johns Hopkins University’s Maryland Food Systems Map (<https://mdfoodsystemmap.org>).

For each surveillance site, an environmental mapping was conducted to characterize the built environment in terms of the number and types of food outlets. Local research teams in each country were trained in administering the instrument on smartphones and tablets. During October 2018 to August 2020, 6-person research teams conducted ground truth data collection surveys, by systematically covering all streets within surveillance sites on foot, following a map in which the site boundary was previously defined by the SAB surveillance data team^1^. The team examined and recorded the presence of any food retailer within each site. To ensure that all streets and neighbourhoods in surveillance sites were covered, we reviewed the map of food outlets on Kobo Toolbox website and Google Maps together with each country team. Also, we deployed quality control checks by sending a second team to the sites to conduct spot checks in randomly selected areas within each site to ensure that all areas were appropriately covered and all relevant food outlets were recorded^2^. Our mapping collected data on geolocations (latitude and longitude) of supermarkets, corner stores (including small grocery and convenient stores), mobile food carts, stationary food carts, and restaurants. Mobile food outlets (stalls, carts, vans, and bikes) were also captured with this tool. Because they are mobile, teams would visit the sites both in the mornings and afternoons, and control checks were performed in days and times that differed from the original data collection timings to ensure the presence of these outlets would be captured in the data.

- 1. ***Food outlet classification***

The categorisation of healthy, unhealthy, and other food retailers was based on products sold within each food retailer as well as international guidelines such as the Retail Food Environment Index (RFEI) and NAICs that classify supermarkets as healthy and fast-food restaurants (FFR) and corner stores as unhealthy^3,4^. Namely, food retailers were classified as healthy if they were supermarkets, unless it was recorded that they sell only unhealthy products such as confectionary, sugar-sweetened beverages (SSBs), or sweet biscuits and no fruit or vegetables in that case they were classified unhealthy, and any other retailer that reports selling fruit or vegetables and no unhealthy products such as confectionary, SSBs and sweet biscuits. Unhealthy food retailers were classified as unhealthy if they were FFR or corner stores, unless they reported selling fruit or vegetables and no unhealthy products such as confectionary, SSBs, and sweet biscuits, in that case these were classified as healthy.

We calculated the density of healthy, unhealthy, plus an “other” category of food outlets. Food environment geolocation data were merged with individual-level data to characterize the density of different food outlet types within 500-m buffer around each participant’s home. We counted the total number of each food outlet and defined density as the share of each food outlet type relative to all food outlets within the 500-m buffer. Food retailers were geospatially linked to individual’s place of residence in ArcGIS Pro.

# **Concentration indices (CI) of healthcare utilisation and advice, and diet-related needs across sex**

| Table S1. Concentration indices of healthcare utilisation and advice, and diet-related needs across sex | | | | | | | | | |
| --- | --- | --- | --- | --- | --- | --- | --- | --- | --- |
|  | Male | | | | Female | | | | Diff. |
|  | N | CI | SE | *p* | N | CI | SE | *p* | *p* |
| **Healthcare utilisation and advice** |  |  |  |  |  |  |  |  |  |
| **Visited healthcare worker** | 78,467 | 0.064 | 0.004 | 0.000 | 124,215 | 0.043 | 0.003 | 0.000 | 0.000 |
| Advice for salt reduction | 78,467 | 0.023 | 0.006 | 0.000 | 124,215 | -0.043 | 0.004 | 0.000 | 0.000 |
| Advice for F&V intake | 78,467 | 0.038 | 0.005 | 0.000 | 124,215 | 0.021 | 0.004 | 0.000 | 0.006 |
| Advice for fat reduction | 78,467 | 0.050 | 0.005 | 0.000 | 124,215 | -0.019 | 0.004 | 0.000 | 0.000 |
| Advice for healthy weight | 78,467 | 0.074 | 0.006 | 0.000 | 124,215 | 0.009 | 0.005 | 0.040 | 0.000 |
| Advice for sugar reduction | 78,467 | 0.037 | 0.006 | 0.000 | 124,215 | -0.052 | 0.005 | 0.000 | 0.000 |
| **Weight-based needs** |  |  |  |  |  |  |  |  |  |
| Underweight | 78,467 | -0.211 | 0.007 | 0.000 | 124,215 | -0.129 | 0.006 | 0.000 | 0.000 |
| Distance to Underweight ^a^ | 36,339 | -0.145 | 0.008 | 0.000 | 40,908 | -0.095 | 0.007 | 0.000 | 0.000 |
| Overweight and obesity | 78,467 | 0.168 | 0.004 | 0.000 | 124,215 | 0.088 | 0.003 | 0.000 | 0.000 |
| Obesity | 78,467 | 0.133 | 0.005 | 0.000 | 124,215 | 0.053 | 0.003 | 0.000 | 0.000 |
| Distance to Overweight ^a^ | 70,211 | 0.078 | 0.003 | 0.000 | 114,514 | 0.019 | 0.002 | 0.000 | 0.000 |
| **Diet-related needs** |  |  |  |  |  |  |  |  |  |
| Suboptimal fat | 44,691 | 0.044 | 0.005 | 0.000 | 71,740 | 0.041 | 0.004 | 0.000 | 0.631 |
| Suboptimal saturated fat | 44,691 | 0.035 | 0.006 | 0.000 | 71,740 | -0.004 | 0.004 | 0.346 | 0.000 |
| Suboptimal free sugars | 44,691 | -0.022 | 0.007 | 0.001 | 71,740 | -0.057 | 0.005 | 0.000 | 0.000 |
| Suboptimal proteins | 44,691 | -0.001 | 0.005 | 0.867 | 71,740 | 0.011 | 0.004 | 0.014 | 0.098 |
| Suboptimal F&V | 44,691 | -0.055 | 0.007 | 0.000 | 71,740 | -0.075 | 0.006 | 0.000 | 0.043 |
| Suboptimal carbohydrates | 44,691 | 0.064 | 0.006 | 0.000 | 71,740 | 0.044 | 0.004 | 0.000 | 0.004 |
| **Environment exposure needs** |  |  |  |  |  |  |  |  |  |
| Healthy Food Outlets ^a^ | 58,984 | 0.034 | 0.003 | 0.000 | 90,714 | 0.008 | 0.002 | 0.001 | 0.000 |
| Unhealthy Food Outlets ^a^ | 58,984 | 0.016 | 0.003 | 0.000 | 90,714 | 0.022 | 0.003 | 0.000 | 0.109 |

Note: we used Wagstaff concentration indices for bounded variables, with fixed scales, and limits from 0 to 1, which zero corresponding to a situation of complete absence (e.g., no healthcare visits).

^a^ We computed the standard concentration index with a zero-fixed lower limit.

# **Concentration indices (CI) of healthcare utilisation and advice, and diet-related needs across countries**

| Table S2. Concentration indices of healthcare utilisation and advice, and diet-related needs across countries | | | | | | | | | | | | |  | |  | |  | |  | |  | |  | |  |
| --- | --- | --- | --- | --- | --- | --- | --- | --- | --- | --- | --- | --- | --- | --- | --- | --- | --- | --- | --- | --- | --- | --- | --- | --- | --- |
|  | Bangladesh | | | | India | | | | | Pakistan | | | | | | Sri Lanka | | | | | | | | Diff. | |
|  | N | CI | SE | *p* | | N | CI | SE | *p* | N | CI | SE | | *p* | | N | | CI | | SE | | *p* | | *p* | |
| **Healthcare utilisation and advice** |  |  |  |  | |  |  |  |  |  |  |  | |  | |  | |  | |  | |  | |  | |
| **Visited healthcare worker** | 70,556 | 0.085 | 0.004 | 0.000 | | 31,644 | 0.140 | 0.006 | 0.000 | 49,059 | -0.038 | 0.006 | | 0.000 | | 51,423 | | -0.023 | | 0.005 | | 0.000 | | 0.000 | |
| Advice for salt reduction | 70,556 | 0.062 | 0.005 | 0.000 | | 31,644 | 0.025 | 0.009 | 0.005 | 49,059 | -0.131 | 0.007 | | 0.000 | | 51,423 | | -0.045 | | 0.006 | | 0.000 | | 0.000 | |
| Advice for F&V intake | 70,556 | 0.065 | 0.005 | 0.000 | | 31,644 | 0.001 | 0.008 | 0.850 | 49,059 | -0.125 | 0.007 | | 0.000 | | 51,423 | | -0.016 | | 0.006 | | 0.011 | | 0.000 | |
| Advice for fat reduction | 70,556 | 0.109 | 0.005 | 0.000 | | 31,644 | 0.018 | 0.008 | 0.026 | 49,059 | -0.141 | 0.007 | | 0.000 | | 51,423 | | -0.028 | | 0.006 | | 0.000 | | 0.000 | |
| Advice for healthy weight | 70,556 | 0.158 | 0.006 | 0.000 | | 31,644 | 0.036 | 0.009 | 0.000 | 49,059 | -0.153 | 0.008 | | 0.000 | | 51,423 | | 0.049 | | 0.007 | | 0.000 | | 0.000 | |
| Advice for sugar reduction | 70,556 | 0.134 | 0.007 | 0.000 | | 31,644 | 0.062 | 0.010 | 0.000 | 49,059 | -0.135 | 0.008 | | 0.000 | | 51,423 | | -0.036 | | 0.006 | | 0.000 | | 0.000 | |
| **Weight-based needs** |  |  |  |  | |  |  |  |  |  |  |  | |  | |  | |  | |  | |  | |  | |
| Underweight | 70,556 | -0.221 | 0.006 | 0.000 | | 31,644 | -0.135 | 0.014 | 0.000 | 49,059 | -0.092 | 0.012 | | 0.000 | | 51,423 | | -0.147 | | 0.010 | | 0.000 | | 0.000 | |
| Distance to Underweight ^a^ | 38,372 | -0.156 | 0.007 | 0.000 | | 8,470 | -0.094 | 0.017 | 0.000 | 11,440 | -0.061 | 0.014 | | 0.000 | | 18,965 | | -0.098 | | 0.011 | | 0.000 | | 0.000 | |
| Overweight and obesity | 70,556 | 0.206 | 0.004 | 0.000 | | 31,644 | 0.086 | 0.007 | 0.000 | 49,059 | 0.055 | 0.006 | | 0.000 | | 51,423 | | 0.096 | | 0.005 | | 0.000 | | 0.000 | |
| Obesity | 70,556 | 0.234 | 0.006 | 0.000 | | 31,644 | 0.050 | 0.007 | 0.000 | 49,059 | 0.042 | 0.005 | | 0.000 | | 51,423 | | 0.044 | | 0.006 | | 0.000 | | 0.000 | |
| Distance to Overweight ^a^ | 61,442 | 0.140 | 0.003 | 0.000 | | 29,734 | 0.022 | 0.003 | 0.000 | 46,209 | 0.013 | 0.003 | | 0.000 | | 47,340 | | 0.022 | | 0.003 | | 0.000 | | 0.000 | |
| **Diet-related needs** |  |  |  |  | |  |  |  |  |  |  |  | |  | |  | |  | |  | |  | |  | |
| Suboptimal fat | 36,228 | 0.060 | 0.006 | 0.000 | | 11,678 | 0.047 | 0.011 | 0.000 | 30,407 | 0.035 | 0.007 | | 0.000 | | 38,118 | | 0.002 | | 0.006 | | 0.733 | | 0.000 | |
| Suboptimal saturated fat | 36,228 | 0.028 | 0.007 | 0.000 | | 11,678 | 0.084 | 0.011 | 0.000 | 30,407 | 0.052 | 0.007 | | 0.000 | | 38,118 | | 0.019 | | 0.006 | | 0.002 | | 0.000 | |
| Suboptimal free sugars | 36,228 | 0.031 | 0.013 | 0.018 | | 11,678 | -0.099 | 0.011 | 0.000 | 30,407 | -0.019 | 0.007 | | 0.011 | | 38,118 | | -0.076 | | 0.007 | | 0.000 | | 0.000 | |
| Suboptimal proteins | 36,228 | -0.016 | 0.006 | 0.011 | | 11,678 | -0.035 | 0.011 | 0.001 | 30,407 | 0.028 | 0.007 | | 0.000 | | 38,118 | | -0.039 | | 0.006 | | 0.000 | | 0.000 | |
| Suboptimal F&V | 36,228 | -0.051 | 0.008 | 0.000 | | 11,678 | -0.154 | 0.017 | 0.000 | 30,407 | -0.053 | 0.010 | | 0.000 | | 38,118 | | -0.082 | | 0.009 | | 0.000 | | 0.000 | |
| Suboptimal carbohydrates | 36,228 | 0.072 | 0.006 | 0.000 | | 11,678 | 0.035 | 0.011 | 0.001 | 30,407 | 0.057 | 0.007 | | 0.000 | | 38,118 | | 0.000 | | 0.006 | | 0.955 | | 0.000 | |
| **Environment exposure needs** |  |  |  |  | |  |  |  |  |  |  |  | |  | |  | |  | |  | |  | |  | |
| Healthy Food Outlets ^a^ | 64,584 | 0.054 | 0.004 | 0.000 | | 18,877 | 0.013 | 0.002 | 0.000 | 32,002 | -0.004 | 0.003 | | 0.189 | | 34,235 | | -0.013 | | 0.004 | | 0.003 | | 0.000 | |
| Unhealthy Food Outlets ^a^ | 64,584 | -0.017 | 0.004 | 0.000 | | 18,877 | 0.003 | 0.003 | 0.298 | 32,002 | -0.050 | 0.003 | | 0.000 | | 34,235 | | -0.007 | | 0.004 | | 0.112 | | 0.000 | |

Note: we used Wagstaff concentration indices for bounded variables, with fixed scales, and limits from 0 to 1, which zero corresponding to a situation of complete absence (e.g., no healthcare visits).

^a^ We computed the standard concentration index with a zero-fixed lower limit.

# **Horizontal inequity index (HII) of healthcare utilisation across sex, using Probit**

| Table S3. Predicted and actual probability of using preventive healthcare in the past 3 years for females (N = 54,079) | | | | | |  |  |
| --- | --- | --- | --- | --- | --- | --- | --- |
|  |  | Probit | | | Need-standardised | | |
| Income quintile | Actual  (1) | Need-predicted  (2) | Difference = predicted - actual | Probit estimates  (3) | | |  |
| Poorest 20% | 0.514 | 0.495 | 1.97% | 0.505 | | |  |
| 2nd poorest 20% | 0.453 | 0.486 | -3.28% | 0.452 | | |  |
| Middle | 0.466 | 0.484 | -1.76% | 0.468 | | |  |
| 2nd richest 20% | 0.457 | 0.477 | -2.05% | 0.465 | | |  |
| Richest 20% | 0.548 | 0.483 | 6.43% | 0.550 | | |  |
|  |  |  |  |  | | |  |
| Mean | 0.488 | 0.485 | 0.28% | 0.460 | | |  |
| **Concentration Index/HHI** | **0.008** | **-0.006** |  | **0.014** | | |  |
| SE | 0.003 | 0.000 |  | 0.003 | | |  |
| t-ratio | 2.917 | -18.804 |  | 4.943 | | |  |

Note: model 1 displays the actual usage of preventive care services; model 2 displays the need-predicted usage of healthcare services, using (i) weight-based needs, (ii) environment risk factors, (iii) dietary needs, and (iv) sex and age differences; model 3 displays the need-standardised probit estimates of predicted preventive care, adjusted by the need variables. All models show actual and predicted values across income quintiles, and the mean values. The horizontal inequity index represents the adjusted concentration indices of each distribution.

| Table S4. Predicted and actual probability of using preventive healthcare in the past 3 years for males (N = 34,129) | | | | | |  |  |
| --- | --- | --- | --- | --- | --- | --- | --- |
|  |  | Probit | | | Need-standardised | | |
| Income quintile | Actual  (1) | Need-predicted  (2) | Difference = predicted - actual | Probit estimates  (3) | | |  |
| Poorest 20% | 0.434 | 0.414 | 1.95% | 0.427 | | |  |
| 2nd poorest 20% | 0.369 | 0.409 | -4.01% | 0.367 | | |  |
| Middle | 0.402 | 0.403 | -0.06% | 0.407 | | |  |
| 2nd richest 20% | 0.303 | 0.400 | -1.73% | 0.390 | | |  |
| Richest 20% | 0.489 | 0.411 | 6.79% | 0.475 | | |  |
|  |  |  |  |  | | |  |
| Mean | 0.414 | 0.485 | 0.67% | 0.414 | | |  |
| **Concentration Index/HHI** | **0.021** | **-0.003** |  | **0.023** | | |  |
| SE | 0.004 | 0.001 |  | 0.004 | | |  |
| t-ratio | 5.000 | -5.103 |  | 5.779 | | |  |

Note: model 1 displays the actual usage of preventive care services; model 2 displays the need-predicted usage of healthcare services, using (i) weight-based needs, (ii) environment risk factors, (iii) dietary needs, and (iv) sex and age differences; model 3 displays the need-standardised probit estimates of predicted preventive care, adjusted by the need variables. All models show actual and predicted values across income quintiles, and the mean values. The horizontal inequity index represents the adjusted concentration indices of each distribution.

# **Horizontal inequity index (HII) of healthcare utilisation across countries, using Probit**

| Table S5. Predicted and actual probability of using preventive healthcare in the past 3 years in Bangladesh (N = 34,118) | | | | | |  |  |
| --- | --- | --- | --- | --- | --- | --- | --- |
|  |  | Probit | | | Need-standardised | | |
| Income quintile | Actual  (1) | Need-predicted  (2) | Difference = predicted - actual | Probit estimates  (3) | | |  |
| Poorest 20% | 0.410 | 0.420 | -1.03% | 0.413 | | |  |
| 2nd poorest 20% | 0.380 | 0.418 | -3.74% | 0.386 | | |  |
| Middle | 0.406 | 0.419 | -1.32% | 0.411 | | |  |
| 2nd richest 20% | 0.438 | 0.425 | 1.21% | 0.436 | | |  |
| Richest 20% | 0.498 | 0.439 | 5.91% | 0.483 | | |  |
|  |  |  |  |  | | |  |
| Mean | 0.424 | 0.424 | 0.01% | 0.424 | | |  |
| **HII** | **0.041** | **0.008** |  | **0.033** | | |  |
| SE | 0.004 | 0.001 |  | 0.004 | | |  |
| t-ratio | 10.100 | 12.547 |  | 8.991 | | |  |

Note: model 1 displays the actual usage of preventive care services; model 2 displays the need-predicted usage of healthcare services, using (i) weight-based needs, (ii) environment risk factors, (iii) dietary needs, and (iv) sex and age differences; model 3 displays the need-standardised probit estimates of predicted preventive care, adjusted by the need variables. All models show actual and predicted values across income quintiles, and the mean values. The horizontal inequity index represents the adjusted concentration indices of each distribution.

| Table S6. Predicted and actual probability of using preventive healthcare in the past 3 years in India (N = 5,323) | | | | | |  |  |
| --- | --- | --- | --- | --- | --- | --- | --- |
|  |  | Probit | | | Need-standardised, no controls | | |
| Income quintile | Actual  (1) | Need-predicted  (2) | Difference = predicted - actual | Probit estimates  (3) | | |  |
| Poorest 20% | 0.680 | 0.691 | -1.18% | 0.698 | | |  |
| 2nd poorest 20% | 0.706 | 0.703 | 0.30% | 0.712 | | |  |
| Middle | 0.675 | 0.713 | -3.79% | 0.671 | | |  |
| 2nd richest 20% | 0.709 | 0.713 | -0.42% | 0.705 | | |  |
| Richest 20% | 0.742 | 0.735 | 0.72% | 0.717 | | |  |
|  |  |  |  |  | | |  |
| Mean | 0.703 | 0.709 | -0.59% | 0.704 | | |  |
| **HII** | **0.011** | **0.012** |  | **-0.001** | | |  |
| SE | 0.005 | 0.002 |  | 0.005 | | |  |
| t-ratio | 2.130 | 6.972 |  | -0.127 | | |  |

Note: model 1 displays the actual usage of preventive care services; model 2 displays the need-predicted usage of healthcare services, using (i) weight-based needs, (ii) environment risk factors, (iii) dietary needs, and (iv) sex and age differences; model 3 displays the need-standardised probit estimates of predicted preventive care, adjusted by the need variables. All models show actual and predicted values across income quintiles, and the mean values. The horizontal inequity index represents the adjusted concentration indices of each distribution.

| Table S7. Predicted and actual probability of using preventive healthcare in the past 3 years in Pakistan (N = 22,798) | | | | | |  |  |
| --- | --- | --- | --- | --- | --- | --- | --- |
|  |  | Probit | | | Need-standardised | | |
| Income quintile | Actual  (1) | Need-predicted  (2) | Difference = predicted - actual | Probit estimates  (3) | | |  |
| Poorest 20% | 0.318 | 0.291 | 2.07% | 0.301 | | |  |
| 2nd poorest 20% | 0.255 | 0.281 | -2.60% | 0.245 | | |  |
| Middle | 0.264 | 0.272 | -0.82% | 0.266 | | |  |
| 2nd richest 20% | 0.279 | 0.266 | 1.29% | 0.287 | | |  |
| Richest 20% | 0.344 | 0.255 | 8.96% | 0.363 | | |  |
|  |  |  |  |  | | |  |
| Mean | 0.289 | 0.274 | 1.54% | 0.289 | | |  |
| **HII** | **-0.000** | **- 0.028** |  | **0.027** | | |  |
| SE | 0.001 | 0.001 |  | 0.007 | | |  |
| t-ratio | -0.036 | -18.482 |  | 3.866 | | |  |

Note: model 1 displays the actual usage of preventive care services; model 2 displays the need-predicted usage of healthcare services, using (i) weight-based needs, (ii) environment risk factors, (iii) dietary needs, and (iv) sex and age differences; model 3 displays the need-standardised probit estimates of predicted preventive care, adjusted by the need variables. All models show actual and predicted values across income quintiles, and the mean values. The horizontal inequity index represents the adjusted concentration indices of each distribution.

| Table S8. Predicted and actual probability of using preventive healthcare in the past 3 years in Sri Lanka (N = 25,969) | | | | | |  |  |
| --- | --- | --- | --- | --- | --- | --- | --- |
|  |  | Probit | | | Need-standardised, no controls | | |
| Income quintile | Actual  (1) | Need-predicted  (2) | Difference = predicted - actual | Probit estimates  (3) | | |  |
| Poorest 20% | 0.614 | 0.624 | -1.00% | 0.597 | | |  |
| 2nd poorest 20% | 0.630 | 0.612 | 1.80% | 0.625 | | |  |
| Middle | 0.581 | 0.603 | -2.24% | 0.584 | | |  |
| 2nd richest 20% | 0.606 | 0.599 | 0.68% | 0.614 | | |  |
| Richest 20% | 0.580 | 0.589 | -0.88% | 0.598 | | |  |
|  |  |  |  |  | | |  |
| Mean | 0.606 | 0.607 | 1.54% | 0.606 | | |  |
| **HII** | **-0.015** | **- 0.011** |  | **-0.004** | | |  |
| SE | 0.003 | 0.001 |  | 0.003 | | |  |
| t-ratio | -4,630 | -21.955 |  | -1.174 | | |  |

Note: model 1 displays the actual usage of preventive care services; model 2 displays the need-predicted usage of healthcare services, using (i) weight-based needs, (ii) environment risk factors, (iii) dietary needs, and (iv) sex and age differences; model 3 displays the need-standardised probit estimates of predicted preventive care, adjusted by the need variables. All models show actual and predicted values across income quintiles, and the mean values. The horizontal inequity index represents the adjusted concentration indices of each distribution.

# **Horizontal inequity index (HII) of healthcare advice across sex and countries, using Probit**

| Table S9. Predicted probability and actual likelihood of receiving salt reduction advice | | | | |
| --- | --- | --- | --- | --- |
|  | N | HII | SE | t-ratio |
| Overall | 88,208 | 0.016 | 0.004 | 3.565 |
| Sex |  |  |  |  |
| Female | 54,079 | 0.003 | 0.006 | 0.512 |
| Male | 34,129 | 0.041 | 0.008 | 5.376 |
| Country |  |  |  |  |
| Bangladesh | 34,118 | 0.050 | 0.007 | 6.810 |
| India | 5,323 | -0.010 | 0.004 | -0.669 |
| Pakistan | 22,798 | -0.054 | 0.010 | -5.251 |
| Sri Lanka | 25,969 | 0.005 | 0.007 | 0.702 |

Note: the horizontal inequity index (HII) represents the concentration indices of each healthcare advice, needs-standardised.

| Table S10. Predicted probability and actual likelihood of receiving F&V consumption advice | | | | |
| --- | --- | --- | --- | --- |
|  | N | HII | SE | t-ratio |
| Overall | 88,208 | 0.037 | 0.004 | 8.813 |
| Sex |  |  |  |  |
| Female | 54,079 | 0.032 | 0.005 | 6.310 |
| Male | 34,129 | 0.044 | 0.007 | 6.316 |
| Country |  |  |  |  |
| Bangladesh | 34,118 | 0.039 | 0.006 | 6.898 |
| India | 5,323 | -0.017 | 0.012 | -1.391 |
| Pakistan | 22,798 | -0.053 | 0.010 | -5.217 |
| Sri Lanka | 25,969 | 0.003 | 0.008 | 0.340 |

Note: the horizontal inequity index (HII) represents the concentration indices of each healthcare advice, needs-standardised.

| Table S11. Predicted probability and actual likelihood of receiving fat reduction advice | | | | |
| --- | --- | --- | --- | --- |
|  | N | HII | SE | t-ratio |
| Overall | 88,208 | 0.022 | 0.004 | 5.014 |
| Sex |  |  |  |  |
| Female | 54,079 | 0.007 | 0.005 | 1.286 |
| Male | 34,129 | 0.051 | 0.007 | 6.821 |
| Country |  |  |  |  |
| Bangladesh | 34,118 | 0.070 | 0.007 | 9.837 |
| India | 5,323 | 0.004 | 0.013 | 0.339 |
| Pakistan | 22,798 | -0.065 | 0.011 | -6.174 |
| Sri Lanka | 25,969 | 0.002 | 0.007 | 0.279 |

Note: the horizontal inequity index (HII) represents the concentration indices of each healthcare advice, needs-standardised.

| Table S12. Predicted probability and actual likelihood of receiving healthy weight advice | | | | |
| --- | --- | --- | --- | --- |
|  | N | HII | SE | t-ratio |
| Overall | 88,208 | 0.026 | 0.006 | 4.738 |
| Sex |  |  |  |  |
| Female | 54,079 | 0.011 | 0.007 | 1.644 |
| Male | 34,129 | 0.057 | 0.010 | 5.982 |
| Country |  |  |  |  |
| Bangladesh | 34,118 | 0.087 | 0.009 | 9.454 |
| India | 5,323 | 0.019 | 0.016 | 1.184 |
| Pakistan | 22,798 | -0.115 | 0.012 | -9.303 |
| Sri Lanka | 25,969 | 0.023 | 0.009 | 2.560 |

Note: the horizontal inequity index (HII) represents the concentration indices of each healthcare advice, needs-standardised.

| Table S13. Predicted probability and actual likelihood of receiving sugar reduction advice | | | | |
| --- | --- | --- | --- | --- |
|  | N | HII | SE | t-ratio |
| Overall | 88,208 | -0.002 | 0.006 | -0.358 |
| Sex |  |  |  |  |
| Female | 54,089 | -0.014 | 0.007 | -2.052 |
| Male | 34,129 | 0.032 | 0.009 | 3.516 |
| Country |  |  |  |  |
| Bangladesh | 34,118 | 0.100 | 0.010 | 9.453 |
| India | 5,323 | -0.001 | 0.018 | -0.071 |
| Pakistan | 22,798 | -0.102 | 0.002 | -28.132 |
| Sri Lanka | 25,969 | 0.006 | 0.008 | 0.699 |

Note: the horizontal inequity index (HII) represents the concentration indices of each healthcare advice, needs-standardised.
